# Adaptive designs for subpopulation analysis optimizing utility functions

**DOI:** 10.1002/bimj.201300257

**Published:** 2014-11-14

**Authors:** Alexandra C Graf, Martin Posch, Franz Koenig

**Affiliations:** 1 Center for Medical Statistics, Informatics, and Intelligent Systems, Medical University of ViennaSpitalgasse 23, 1090, Vienna, Austria; 2 Competence Center for Clinical Trials, University of BremenLinzer Strasse 4, 28359, Bremen, Germany

**Keywords:** Adaptive design, Enrichment design, Hypothesis selection, Sample size reallocation, Utility function

## Abstract

If the response to treatment depends on genetic biomarkers, it is important to identify predictive biomarkers that define (sub-)populations where the treatment has a positive benefit risk balance. One approach to determine relevant subpopulations are subgroup analyses where the treatment effect is estimated in biomarker positive and biomarker negative groups. Subgroup analyses are challenging because several types of risks are associated with inference on subgroups. On the one hand, by disregarding a relevant subpopulation a treatment option may be missed due to a dilution of the treatment effect in the full population. Furthermore, even if the diluted treatment effect can be demonstrated in an overall population, it is not ethical to treat patients that do not benefit from the treatment when they can be identified in advance. On the other hand, selecting a spurious subpopulation increases the risk to restrict an efficacious treatment to a too narrow fraction of a potential benefiting population. We propose to quantify these risks with utility functions and investigate nonadaptive study designs that allow for inference on subgroups using multiple testing procedures as well as adaptive designs, where subgroups may be selected in an interim analysis. The characteristics of such adaptive and nonadaptive designs are compared for a range of scenarios.

## 1 Introduction

Technical methods to investigate the genetic heterogeneity of patients have improved rapidly. In the development of targeted therapies there is an increasing interest in clinical trials investigating predictive biomarkers (Beckman et al., 2011; Ziegler et al., 2012) that explain the genetic diversity of patients therapeutic response.

Subgroup analyses in clinical trials to assess the consistency of a treatment effect in different subpopulations defined by genetic markers have often been considered as exploratory analysis only and confirmatory claims on the treatment effect were made only for the total trial population. In recent years clinical trials with more complex objectives, which allow one to confirm a treatment effect in the overall population as well as only in a subpopulation, have raised more and more attention.

In the development of targeted therapies with prior evidence that the treatment effect may be stronger (or only present) in a subgroup defined by a biomarker, one faces several design options when planning a clinical trial. The trial can be performed either in the biomarker positive subgroup only or in the full population (Maitournam et al., 2005; Mandrekar and Sargent, 2009a,b; Freidlin et al., 2013). In the latter case, a multiple testing procedure can be preplanned to allow one to test for a treatment effect in the subgroup as well as in the full population (Song and Chi, 2007; Alosh and Huque, 2009; EMA, 2010; Millen et al., 2012). A third option, that may be attractive in situations with considerable uncertainty left on the treatment effect in the biomarker negative subgroup, are adaptive designs that allow one to enrich the study population after an interim analysis. In a first stage patients are recruited from the full population. In the interim analysis, the trial population may be adapted based on the observed treatment effects in the subgroup. The trial continues either in the full population or in a subpopulation only. To control the type I error rate adjusting for the adaptive choice of populations as well as the multiplicity arising from the testing of subgroups, combination tests (Bauer and Koehne, 1994; Bauer and Kieser, 1999; Bretz et al., 2009) and the conditional error rate principle (Mueller and Schaefer, 2001, 2004) have been proposed (Brannath et al., 2009; Jenkins et al., 2011; Friede et al., 2012; Stallard et al., 2014; Wang et al., 2007). These approaches base the test decision on data from the first and the second stage of the trial. Different decision rules to select the population for the second stage have been considered, ranging from simple rules based on differences of z-statistics (Kelly et al., 2005; Friede et al., 2012) to Bayesian decision tools (Brannath et al., 2009).

All the above approaches require that the subpopulation is prespecified which is the most common scenario in a confirmatory setting. However, also more general approaches have been proposed, that allow one to search for predictive biomarkers to define a subgroup based on the first stage data (Freidlin and Simon, 2005; Jiang et al., 2007; Mehta et al., 2009). With these approaches, however, the statistical test for the identified subgroup uses the second stage data only. Another generalization are trial designs for settings with more than one subpopulation (Magnusson and Turnbull, 2013).

It has been shown that adaptive designs may lead to superior statistical power compared to fixed sample designs, where power is usually defined as the power to reject at least one false null hypothesis (Wang et al., 2009; Boessen et al., 2013). In a setting where multiple hypotheses are tested, however, this may not be the only operating characteristic of interest. Other power definitions, such as the average power, or the power to reject all null hypotheses have been proposed (Stallard et al., 2009; Bretz et al., 2009). A limitation of the latter power concepts is that they are symmetric in all tested hypotheses and therefore cannot appropriately reflect the objectives in the setting of subgroup analyses where the consequences of inferences on subgroups and the full populations may substantially differ.

Inference on subpopulations is challenging because different types of risks need to be accounted for: On the one hand, disregarding a relevant subpopulation one may miss a treatment option due to a dilution of the treatment effect in the full population. Furthermore, even if the diluted treatment effect can be demonstrated in an overall population, it is not ethical to treat patients that do not benefit from the treatment, when they can be identified in advance. On the other hand, selecting a spurious subpopulation increases the risk to erroneously conclude that a treatment is efficacious (inflating the type I error rate), or may wrongly lead to restricting an efficacious treatment to a too narrow fraction of a potential benefiting population. The latter can not only lead to a reduced revenue from the drug, but is also unfavorable from a public health perspective. Instead of focusing on power definitions, we quantify these risks with utility functions and investigate the characteristics of adaptive and nonadaptive study designs that allow for confirmatory inference on subgroups controlling the family wise type I error rate. In addition, we derive optimized adaptive designs that maximize expected utilities by optimizing the first stage sample size and decision thresholds for the selection of subgroups.

The paper is structured as follows. In Section 2 we discuss fixed sample designs and compare their performance based on their expected utility. In Section 3 we assess adaptive approaches based on expected utilities and use simulation results to identify optimized designs for a range of scenarios. The findings and extensions of the approach are discussed in Section 4.

## 2 Fixed sample design

Consider a clinical trial where a treatment is compared to a control in a parallel group design and a subpopulation *S* (e.g. based on a biomarker) is investigated. Let 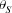 (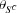) denote the true difference in means (control versus experimental arm) of a normally distributed endpoint in the subpopulation *S* and its complement 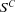. Then the treatment effect in the full population is given by 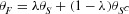, where λ denotes the prevalence of subpopulation *S*. For this setting we consider two design options to plan a fixed sample clinical trial:

- **Stratification design:** Patients are recruited from the full population and hypotheses tests for both populations are performed, testing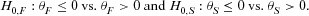  Due to performing two tests (for *F* and *S*), a multiplicity adjustment is performed to control the family wise type 1 error rate at a prespecified level α.
- **Enrichment design:** Patients are recruited from the subpopulation only (achieving the same overall sample size as in the stratified design) and efficacy is tested only in the subpopulation, testing
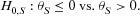

While both designs allow one to test 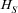, the stratification design additionally tests for a treatment effect in the full population. However, assuming the same total sample size *n* per treatment group, the enrichment design includes a larger number of patients from subpopulation *S*.

In the following we consider a parallel group comparison for the means of two normal-distributions with common known variance σ. The effect 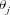 is assumed to be the mean difference between treatment and control for 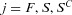. In the enrichment design, 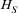 is tested using a z-test with test statistics 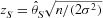 where 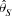 is the observed effect estimate using the total sample size *n* per group, assuming groups of equal size and a common known variance σ^2^. In the stratification design 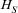 is tested with a z-test with test statistics 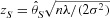 and 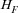 is tested with a stratified z-test 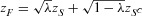 where 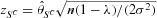 is the test statistic of the complement. Correction for multiplicity in the stratification design is performed using the Hochberg test (Hochberg, 1988; Simes, 1986). For both designs the total per treatment group sample size *n* is chosen such that in the stratified design a standardized effect size in the full population of 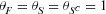 can be detected at level 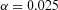 and the power to reject at least one of the two hypotheses 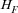 or 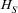 is about 0.8, given a prevalence of 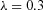.

### 2.1 Power considerations

The power to reject any of the two hypotheses depends on the unknown true effect sizes 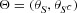 as well as the prevalence λ of the subgroup. In a setting where a targeted therapy is developed, there is uncertainty whether 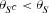. Note that the case 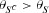 is not considered in the power calculations as we assume that it is ruled out for scientific reasons. For the given setting the enrichment design (recruiting only patients in *S*) always leads to the highest power to reject at least one null hypothesis: if 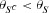 the enrichment design has larger power due to the larger effect and the larger sample size for the subgroup *S* as compared to the stratification design, where the sample size of *S* is 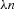. Note also that there is a dilution of the treatment effect in the full population for the stratification design. If 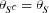 the enrichment design has a larger power because the stratification design is using an adjustment for multiple testing due to performing two tests (for *F* and *S*). Thus, if in truth 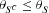 (which is the underlying assumption for the consideration of the subgroup), regarding the power to reject any hypothesis the enrichment design is always preferable.

However, it appears that the power to reject any null hypothesis does not appropriately reflect the objectives in this setting. The enrichment design allows one to demonstrate a treatment effect in the subpopulation only. While revenues are complex and multifactorial, one would expect that this leads to a lower gain for the sponsor simply due to the smaller size of the population the drug can be marketed to after regulatory approval. Especially in an indication where the market is saturated and competitor drugs are already approved, the loss in the number of potential patients cannot be compensated by higher prices because the per patient price paid by reimbursement bodies is restricted by the price of competitor products. More importantly, the restriction to a subgroup only in an enrichment design may raise ethical concerns because patients that potentially may benefit from the treatment are excluded. To account for these aspects, we consider an approach based on utility functions.

### 2.2 Utility functions for decisions on subgroups

Considering the power to reject any null hypotheses implies that the outcomes “reject 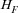
” and “reject 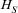
” are equally desirable. However, the gain for the sponsor as well as the gain from a public health perspective depends on which hypothesis is actually rejected. To quantify the gain, we propose utility functions that assign different gains to different outcomes of the test. As examples, we consider two simple utility functions, in the following denoted by “sponsor view” and “public health view”. While these utility functions are somewhat simplistic and cannot cover all aspects of utilities in the considered scenarios, they better formalize the key components than traditional power considerations and allow for a systematic evaluation of study designs under different perspectives.

For the “sponsor view” utility function we assume that when showing a treatment effect in the full population, that is 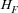 is rejected, a gain 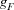, is achieved. If the treatment effect is shown in the subpopulation only, that is 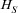 is rejected only, a smaller (or equal) gain 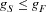 is achieved because from the sponsor's perspective, demonstrating a treatment effect in a smaller population implies a smaller market. Furthermore, we assume the gain 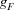 achieved if efficacy is demonstrated in the full population, does not depend on whether the treatment is in truth effective or not. If none of the two hypotheses is rejected, the gain is 0. Thus, the sponsor's view utility function is given by


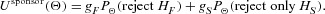
(1)

Note that the utility under the “sponsor view” depends on the test decisions only but not on the true effect in the considered populations. The “sponsor view” is motivated by the work of Beckman et al. (2011) who suggest to use Phase 2 data to decide whether performing an (adaptive) enriched study or not. In contrast, the “public health view” utility function depends on both, the test decisions and the true effects in the subpopulations. We define,


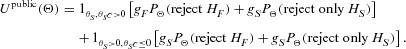
(2)

The public health view assigns the gain of 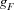 if 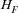 is rejected and there is a homogeneous treatment effect in 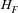 such that the treatment is effective in *S* and 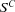. If the treatment is effective in *S* only, the gain is assumed to be equal to 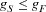 regardless if 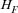 or 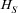 is rejected. This reflects the fact, that only the patients in the subset *S* will actually benefit from the treatment. For 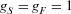 the two utility functions *U*^sponsor^ and *U*^public^ are both equal to the power of rejecting at least one of the two hypotheses (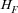 or 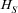). Note that we do not explicitly include costs in the utility functions. However, we restrict the comparison of trial designs to designs with equal overall sample size. Assuming the trial costs to be proportional to the sample size, we therefore compare only trial designs with the same costs. Furthermore, without restricting generality we normalize the gains by setting 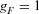.

**Figure 1:**
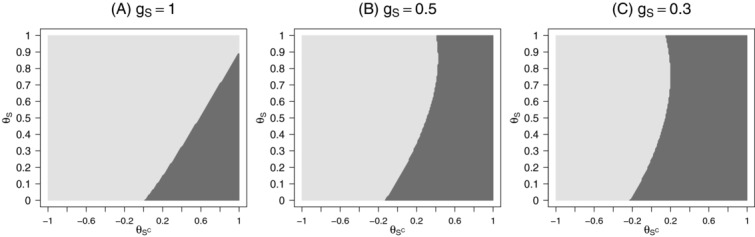
Subsets in the 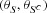-plane where the enrichment (light-gray) or the stratification design (dark-gray) are achieving the highest expected utility for the sponsor view, setting 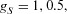 and 0.3 (panels A, B, C), and the prevalence was set to 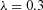.

Which of the two design options, the stratification or the enrichment design, is preferable in terms of utility depends on the effect sizes of the subpopulation, 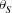, and the complement, 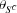, the prevalence, λ and the gain 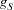. Figure 1 shows the subsets in the 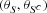-plane where the stratification or the enrichment design lead to a higher expected utility. Values are given under the sponsor view for different 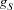 assuming 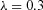. Note, that if 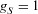 or 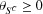 the public health view is equal to the sponsor view leading to the same preferable designs. For 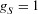 (i.e. the utility functions are equal to the power of rejecting any hypothesis) the stratification design is only preferable if 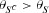, however, this is a parameter constellation which is typically not considered plausible if a targeted therapy is investigated. With decreasing 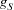 (i.e. a smaller gain if efficacy is shown in the subgroup only) the parameter range where the stratification design is preferable increases. For larger positive 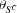 the stratification design is leading to a higher expected utility due to the larger chance of rejecting 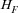 and therefore achieving the gain 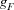. However, also for small negative 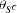 and small 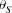 the stratification design is preferable under the sponsor view. This is in contrast to the public health view, where for 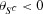, always the enrichment design is preferable. For small positive 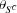 the stratification design is optimal for very small and very large 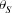 but not for intermediate effect sizes: If both 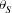 and 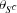 are small, the power of both the enrichment and the stratification design is close to the significance level, but the stratification design leads to a larger gain. For intermediate 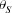 the effect size in the full population is too diluted such that the loss in power of the stratification design cannot be compensated by the increased gain if 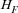 is rejected. For very large 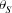 however, the treatment effect in the full population (driven mainly by the subgroup) is large enough to guarantee sufficient power to test 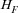 and the stratification design has a higher utility.

Assessing the utility of clinical trial designs under specific assumptions on the efficacy parameters can be a useful tool when assessing different design options, but it does not take into account uncertainty in the prior knowledge on effect sizes. To account for this uncertainty we consider a Bayesian approach to quantify expected utility. To this end we consider a prior assuming that the treatment is effective in the subpopulation but that there is uncertainty about the treatment effect in the complement. For simplicity we restrict the investigations to a two point prior reflecting the scenarios where the treatment either has an effect of 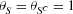 in both *S* and 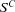 or an effect of 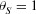 in *S* but no effect (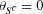) in the complement. Thus, the prior is defined by a single probability π that the treatment is efficacious in *S* and 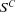.

**Figure 2:**
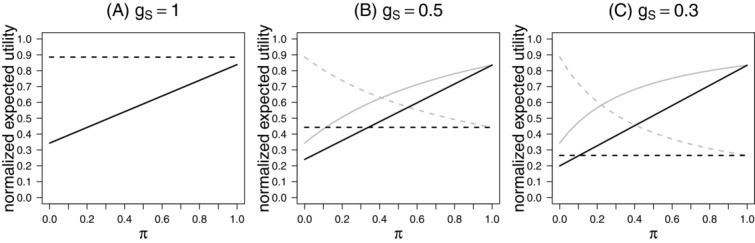
Expected normalized utility for the fixed sample design as a function of the prior probability π for different gains 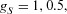 and 0.3 (panels A, B, C) setting 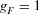. Expected normalized utility is shown for the public health view (gray lines) and the sponsor view (black lines) for the stratification design (solid lines) and the enrichment design (dashed lines). The prevalence was set to 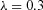.

Figure 2 shows the normalized expected utility 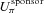 (sponsor view) as well as 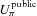 (public health view) as a function of the prior π for 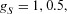 and 0.3, assuming a prevalence of 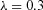. For each 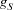 and prior π the utilities are normalized by the corresponding maximum achievable utility (assuming all false null hypotheses can be rejected with probability 1). For the sponsor view the maximum utility is 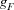, such that the normalized utility is given by 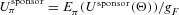. For the public health view the maximal achievable utility depends on the prior π and is given by 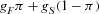, such that the normalized utility is 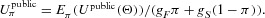 The normalized expected utility can then be interpreted as the proportion of the expected utility that is achieved compared to the maximum achievable utility under a certain prior and utility function. Note that the normalization has no impact on the selection of the preferable trial design for a specific utility function.

As noted above, for 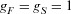 the utilities 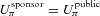 are equal to the power of rejecting at least one hypothesis and the enrichment design (dashed line) has a larger power over all prior probabilities as compared to the stratification design (solid line). The situation changes, however, if the gain 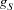 for rejecting 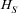 is smaller than 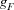. Note again that 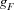 was set to 1.

While for small π (i.e. a strong prior evidence that the treatment works in the subgroup only) the enrichment design is still leading to a higher expected utility compared to the stratification design, for larger π the stratification design is preferable. The smaller 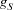 the larger the area where the stratification design is preferable in terms of the given utility functions. For the sponsor view the range of prior distributions where the stratification design is preferable is larger than for the public health view and this difference increases with decreasing 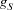.

## 3 Adaptive approach

If there is prior evidence of a treatment effect in a certain subpopulation but little or no knowledge on the treatment effect in its complement, a further design option is an adaptive approach which is an intermediate strategy between the enrichment and the stratification design (Bretz et al., 2009; Brannath et al., 2009; Chen and Beckman, 2009; Beckman et al., 2011; Sargent and Madrekar, 2013; Freidlin and Korn, 2014). In adaptive designs the treatment effects are estimated in an interim analysis and the design of the remaining part of the trial maybe modified. Consider, for example, a trial that starts in an overall unselected population. If the treatment effect estimate in the biomarker negative subpopulation crosses a futility threshold in an interim analysis, accrual maybe restricted to the biomarker positive subgroup. Such designs have been proposed and formalized for ethical and efficiency reasons to minimize the number of patients that are treated with a nonefficacious treatment. Assume now, that an interim analysis is performed after a first stage. An overall sample size *n* per group was preplanned and the interim analysis is performed after 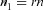 observations per group (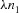 observations in the subpopulation). Based on the interim results, it is decided to continue only with *S* (testing only for *S*) or to continue with *F* (testing *F* and *S*), that is the first stage data is used to choose the second stage population. The efficacy of the treatment is then demonstrated using data of both stages.

### 3.1 Adaptive closed test

To control the family wise type I error rate in the strong sense for the given adaptive enrichment design, the closure principle (Marcus et al., 1976) using adaptive combination tests as local tests can be applied (Bauer and Kieser, 1999; Hommel, 2001; Bretz et al., 2009). To apply the closure principle, local level α tests for the elementary hypotheses 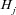, 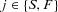 and the intersection hypothesis 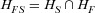 have to be defined. Then the closure test rejects an elementary hypothesis 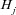, 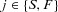 controlling the family wise type I error rate if the intersection hypothesis 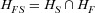 and 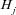 can be rejected at local level α. In the adaptive setting as local level α tests combination tests are performed. To this end, a combination function 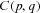 is defined, which is a function of a first stage *p*-value *p* and a second stage *p*-value *q*, where the latter is computed from the second stage data only. The combination test rejects if 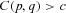, where the critical value *c* is calculated such that for independent and uniformly distributed *p*-values 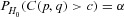. In the adaptive enrichment design we have two options (say, options A and B) at the interim analysis: If the trial continues in *F* (option A) the local combination test rejects 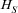 if 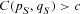, and 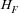 if 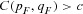, where 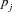 and 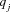, 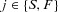 are the elementary *p*-values of the respective tests based on the first and second stage data. If the trial continues in *S* only (option B), we formally set 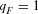 and 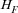 is retained. To test the intersection hypothesis 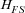 we again apply a combination test. As first stage test we use the Hochberg test (Hochberg, 1988) such that the first stage *p*-value 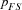 is given by 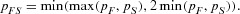 The choice of the second stage test depends on the adaptation decisions in the interim analysis. If the trial is continued with *F* (option A), the second stage test is again a Hochberg test and the second stage *p*-value 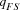 is defined as above replacing 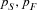 by 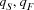. If the trial is continued with *S* only (option B), we set 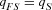. Then, the combination test rejects 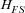 at local level α if 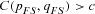. Thus, the adaptive closed test rejects 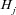, 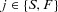 if 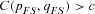 and 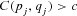.

Note that the population selection rule at interim may depend on the interim data and on external data in any way. The selection rule needs not to be specified in detail. Furthermore, we may apply sample size adaptations based on unblinded interim data. Using the adaptive closed test, the family wise type I error rate is controlled in the strong sense (see e.g. Bretz et al., 2009).

### 3.2 Optimized adaptive designs

Consider an adaptive design where the decision on continuing with the full- or the subpopulation is based on the observed effect size of the treatment in the complement 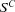. If the first stage *p*-value 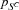 for the test of the treatment effect in the complement 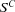 is smaller than a threshold α_0_ (i.e., there is a promising effect in 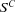), the study continues with the full population (option A in Section 3.1), with a second-stage sample-size 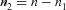 per group including a sample size of 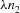 from the subpopulation. If 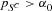, indicating that there is no promising effect of the treatment in the complement, the trial will be continued with the subpopulation only (option B in Section 3.1). Here, *n*_2_ patients per group of the subpopulation only are recruited in the second stage. Note that such a design incorporates two types of adaptation at the same time: If the trial continues with the subpopulation only, the hypothesis 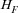 is dropped and the sample size is reallocated by increasing the sample size for the remaining hypothesis 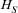.

As combination function we use the weighted inverse normal combination function approach of Lehmacher and Wassmer (1999) setting


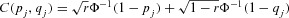


for 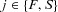, where 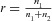 is the weight of the first stage test statistics and 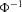 the quantile of the standard normal distribution. Setting 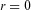 (and therefore 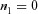) and 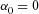 the adaptive design reduces to the enrichment design (ii) in Section 2011, that is the fixed sample trial in the subpopulation only. Setting 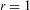 (i.e. 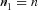) and 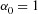, the adaptive design is equal to the stratification design (i) in Section 2011, that is a fixed sample trial in the full population, testing both hypothesis 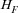 and 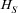. For 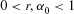 the design is adaptive with a first stage corresponding to a stratification design and a second stage corresponding to the stratification or enrichment design depending on the interim decision.

In the comparison below, optimized adaptive designs were considered, optimized in the parameters *r* (and thus 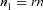 determined by *r*) and α_0_ with respect to the expected utilities 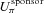 and 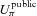. Optimization is performed by simulating the trial designs for a grid of *r* and α_0_ values with 100,000 simulation runs per grid point and selecting the design with the highest expected utility. The grid ranged from 0 to 1 in steps of 0.001. The stage wise *p*-values are computed based on z-tests and the overall per group sample size *n* is chosen as in Section 2011.

**Figure 3:**
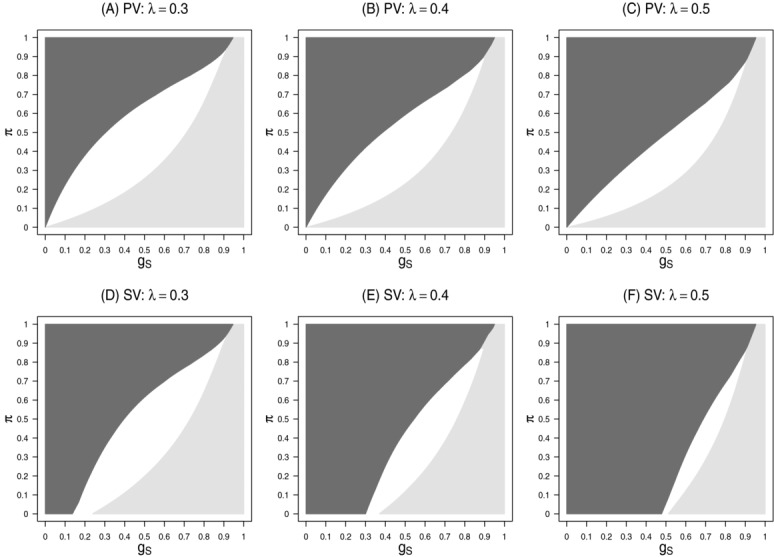
Subsets in the 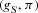-plane where the enrichment design (light-gray), the adaptive design (white) and the stratification design (dark-gray) show the largest expected utility for the public health view (first row) and the sponsor view (second row). The prevalence is set to 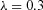 (first column), 0.4 (second column), and 0.5 (third column).

Figure 3 shows the subsets in the 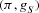-plane where the stratification, enrichment or adaptive designs have the highest expected utility. For the adaptive designs, the optimized adaptive design with optimal parameters *r* and α_0_ are chosen. The results are given for the public health and sponsor view utility functions.

For both utility functions, for large 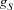 and small π the enrichment design is leading to the largest expected utility while for small 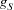 and large π the stratification design is preferable. Only for intermediate values of 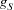 and *r* an adaptive design is preferable. With increasing prevalence λ, the range of scenarios where the adaptive design is preferable decreases. This holds for both utility functions. Note that for the sponsor view utility function the range of scenarios where the adaptive design is preferable is smaller than for the public health view utility function. For the sponsor view, the area where the stratification design is preferable is larger than for the public health view, because in the latter a rejection of 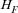 (whose test has the highest power in the stratification design) entails an additional gain only if the treatment is also effective in the complement of *S*.

**Figure 4:**
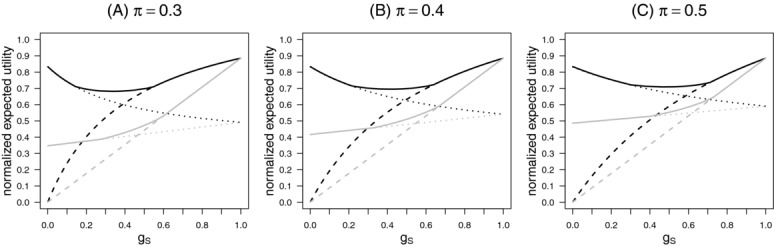
Normalized expected utility for the optimal design (solid lines), the stratification design (dotted lines), and the enrichment design (dashed lines) as a function of the gain 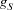 for prior probability 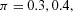 and 0.5 (panels A, B, C), separately for the public health view (black lines) and the sponsor view (gray lines). The prevalence λ was set to 0.3.

Figure 4 shows the normalized expected utility for the optimal design (solid lines), the stratification design (dotted lines), and the enrichment design (dashed lines) as a function of the gain 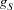 for prior probability 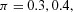 and 0.5 separately for the public health view (black lines) and the sponsor view (gray lines). For the sponsor view the advantage of the adaptive design may be small as compared to the fixed sample enrichment or stratification design. For the public health view, the gain in utility is larger, however decreasing with increasing π.

Table 1 shows for several values of the gain 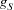 and the prior π the optimal design parameters *r* and α_0_ as well as the corresponding normalized utility and the normalized utility of the enrichment and the stratification design for the public health and sponsor view. The prevalence λ was set to 0.3. For increasing 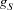, the threshold α_0_ is decreasing, reflecting that for larger 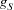 the adaptive design is approximating the enrichment design. For increasing prior probability π, α_0_ is increasing, reflecting that for larger π the stratification design is preferable.

**Table 1:** Optimal design parameters *r* and α_0_, the corresponding normalized utility as well as the normalized utility of the enrichment and the stratification design for the public health and sponsor view for several values of the gain 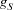 and the prior π. The prevalence was set to 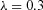

		Public health view					Sponsor view				
			
		Optimal			Fixed design		Optimal			Fixed design	
		design			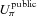		design			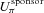	
					
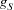	π	*r*	α_0_	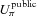	Enrichment	Stratification	*r*	α_0_	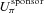	Enrichment	Stratification
0.2	0.3	0.34	0.48	0.70	0.40	0.68	1.00	1.00	0.38	0.18	0.38
0.2	0.4	1.00	1.00	0.72	0.34	0.72	1.00	1.00	0.44	0.18	0.44
0.2	0.5	1.00	1.00	0.75	0.30	0.75	1.00	1.00	0.51	0.18	0.51
0.3	0.3	0.31	0.32	0.68	0.52	0.63	0.34	0.48	0.39	0.27	0.39
0.3	0.4	0.30	0.41	0.70	0.46	0.68	1.00	1.00	0.45	0.27	0.45
0.3	0.5	1.00	1.00	0.72	0.41	0.72	1.00	1.00	0.52	0.27	0.52
0.4	0.3	0.26	0.24	0.68	0.61	0.60	0.24	0.40	0.43	0.35	0.40
0.4	0.4	0.31	0.32	0.70	0.55	0.65	0.32	0.46	0.48	0.35	0.47
0.4	0.5	0.30	0.41	0.71	0.51	0.69	0.34	0.48	0.53	0.35	0.53
0.5	0.3	0.18	0.19	0.70	0.68	0.57	0.21	0.26	0.47	0.44	0.42
0.5	0.4	0.26	0.24	0.70	0.63	0.62	0.24	0.34	0.51	0.44	0.48
0.5	0.5	0.31	0.32	0.71	0.59	0.67	0.30	0.41	0.55	0.44	0.54
0.6	0.3	0.00	0.00	0.74	0.74	0.55	0.00	0.00	0.53	0.53	0.43
0.6	0.4	0.18	0.19	0.71	0.70	0.60	0.20	0.24	0.55	0.53	0.49
0.6	0.5	0.26	0.24	0.72	0.66	0.65	0.21	0.34	0.59	0.53	0.55
0.7	0.3	0.00	0.00	0.78	0.78	0.53	0.00	0.00	0.62	0.62	0.45
0.7	0.4	0.00	0.00	0.76	0.76	0.58	0.00	0.00	0.62	0.62	0.50
0.7	0.5	0.12	0.15	0.73	0.73	0.63	0.14	0.20	0.63	0.62	0.56

### 3.3 A utility function penalizing efficacy claims for too large populations

In settings where the treatment is effective in *S* but not in 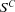, the public health utility function 2014 specifies the same gain 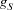 for the rejection of 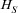 as for the rejection of 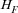. However, in scenarios where the treatment entails a safety risk or if the cost of the treatment is taken into account, a utility that penalizes efficacy claims for a too large population may be more appropriate. To this end we introduce a further parameter 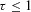 and define


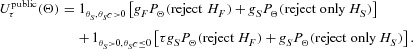
(3)

Setting 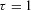 gives the utility function 2014 and implies that claiming efficacy for a too large population (population *F* when the treatment is efficacious in *S* only) is not penalized in the utility function. Setting 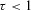 the utility function assigns a lower utility to the rejection of 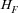 than 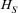 in the setting where the treatment is effective in *S* only. If we assume that the cost to treat a patient in 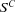 (where the treatment is not efficacious) is equal to the gain to treat a patient in *S* (where the treatment is efficacious), the utility assigned to the event that 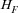 is rejected when the treatment is only efficacious in *S*, is given by 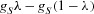. This corresponds to 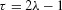 in 2013.

**Figure 5:**
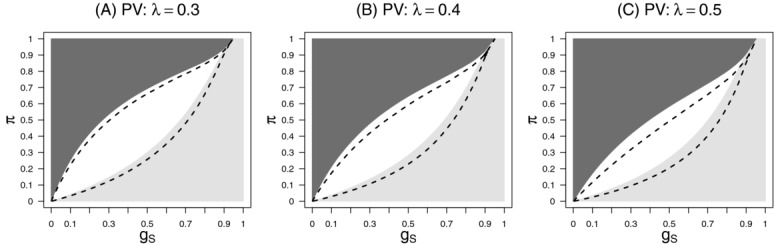
Subsets in the 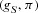-plane where the enrichment design (light-gray), the adaptive design (white), and the modified stratification design with optimized consistency bound (dark-gray) show the largest expected utility for the public health view when optimizing the consistency boundary. The prevalence is set to 0.3 (panel A), 0.4 (panel B), and 0.5 (panel C) with 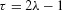. For comparison, the dashed lines give the corresponding area boundaries for 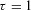 and the testing procedure without consistency boundary, that is, the corresponding areas in Fig. 3, first row.

To optimize the trial design for the public health utility function when 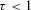 we extend the adaptive test by introducing a consistency boundary *c* such that 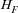 is rejected in the final analysis if the adaptive closed test rejects 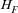 and additionally 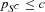, where 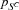 denotes the *p*-value for the comparison of means in 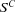 pooled over both stages. Thus, 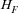 can only be rejected if also a minimum efficacy in 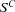 is observed. For a given prior, prevalence, and parameters 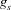 and τ we optimized the consistency boundary *c* together with α_0_ and *r* to maximize the utility function 2013. We determined the optimal design parameters by simulating the expected utility over a grid of the parameters 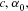 and *r* ranging from 0 to 1 in steps of 0.01. Figure 5 shows that for 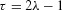 the set of priors π and gains 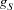 where the enrichment design is best is larger and the set where the stratification design is best is smaller compared to the case 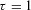. Table 2 gives the optimal adaptive designs and its normalized utilities compared to the enrichment and the stratification design for several values of gains 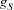 and priors π. Note that the adaptive design with 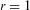 and 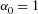 corresponds to a stratified design where 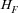 is rejected only if the *p*-value of the test of 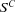 is lower than *c*. Such modified stratification designs are included in the dark-gray area in Fig. 5.

**Table 2:** Optimal design parameters *r*, α_0_, and *c*, the corresponding normalized utility as well as the normalized utility of the enrichment and the stratification design for the modified public health view utility with 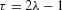 for several values of the gain 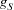 and the prior π. The prevalence was set to 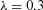

		Optimal design				Fixed design 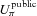	
			
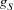	π	*r*	α_0_	*c*	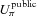	Enrichment	Stratification
0.2	0.3	0.39	0.34	0.11	0.66	0.40	0.64
0.2	0.4	0.44	0.41	0.14	0.69	0.34	0.69
0.2	0.5	1.00	1.00	0.18	0.73	0.30	0.73
0.3	0.3	0.30	0.28	0.08	0.65	0.52	0.59
0.3	0.4	0.37	0.33	0.10	0.67	0.46	0.64
0.3	0.5	0.41	0.40	0.14	0.69	0.41	0.69
0.4	0.3	0.23	0.20	0.06	0.66	0.61	0.55
0.4	0.4	0.28	0.29	0.08	0.67	0.55	0.61
0.4	0.5	0.34	0.33	0.10	0.69	0.51	0.66
0.5	0.3	0.00	0.00	0.00	0.68	0.68	0.52
0.5	0.4	0.23	0.20	0.07	0.68	0.63	0.58
0.5	0.5	0.28	0.27	0.08	0.69	0.59	0.64
0.6	0.3	0.00	0.00	0.00	0.74	0.74	0.51
0.6	0.4	0.00	0.00	0.00	0.70	0.70	0.56
0.6	0.5	0.21	0.20	0.07	0.70	0.66	0.61
0.7	0.3	0.00	0.00	0.00	0.78	0.78	0.49
0.7	0.4	0.00	0.00	0.00	0.76	0.76	0.54
0.7	0.5	0.00	0.00	0.00	0.73	0.73	0.60

## 4 Discussion

In this manuscript we considered the problem of designing a clinical trial in the setting where only a subgroup of patients may benefit from a treatment. To compare different design options we propose to quantify the achieved gains resulting from the different outcomes of a trial by utility functions. Then, different trial designs can be compared with regard to the expected utility. While the considered clinical trials designs are based on frequentist hypothesis tests, the evaluation of the expected utility of the trials follows a Bayesian approach, assuming a prior distribution on the efficacy parameters.

Quantifying the expected utility of different trial designs is a complex task. In general, the utility will depend on the outcome of the clinical trial as well as external factors and will differ between different stakeholders as companies, patients, and society. The utility functions considered in this paper cover important basic factors that determine the utility and give a transparent framework that allows to understand the impact of key parameters on the utilities of different clinical trial designs. To include additional factors into the model, the utility functions can be extended in several ways. A generalization is to allow the utility functions to depend on the effect sizes. For the public health view the actual effect sizes are most relevant, while for the sponsor view, the observed effect sizes as considered in Posch and Bauer (2013) may be more important. We also made the simplifying assumption that the cost of the trial is proportional to the total sample size, such that by comparing designs with the same total sample size, the costs need not be explicitly included in the utility function to compare different design options. Extending the utility function, one could account for situations where the restriction of the recruitment to a subpopulation increases the costs and duration of a trial and take into account that more complex clinical trial designs are more costly to implement. Furthermore, while we focused on simple two point prior distributions, the approach can be easily extended to more complex priors for the efficacy parameters. Another extension of the proposed approach is to explicitly include costs for false positive decisions in the utility function. We considered hypotheses testing procedures that control the type I error rate at a prespecified level (usually 2.5%). Including costs for false positive decisions, the optimization can be extended to determine optimal significance levels that maximize expected utility by balancing type I and type II errors leading to a classical Bayesian decision problem. Such an approach may gain relevance as regulators recently discussed that excessive risk aversion may not be in the best interest of patients and public health (Eichler et al., 2013) and there is a need to balance false positive and false negative decisions. Advanced statistical expertise will be required to implement such methods in regulatory decision making (Bauer and Koenig, 2014).

The optimization results show that the optimal trial design depends sensitively on the weights of the prior distribution and on the parameters 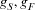 that quantify the different gains for rejection of 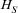 and 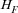. For the sponsor view utility function, these parameters may be determined by the net present value of the treatment which depends, among many other factors, on the prevalence of the population it is marketed to. For example, Beckman et al. (2011) use a Bayesian decision analysis approach after Phase 2 data are available to decide if the Phase 3 trial should be enriched, stratified in the full population, adaptive or better not be conducted. They suggest that the actual utilities of falsely or truly rejecting 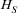 or 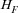 should be determined by the drug development team, and therefore corresponds to the sponsor view. For the public health view the quantification of the utility of different outcomes may be measured in overall quality-adjusted life years, or a score that additionally takes the costs for the treatment into account (Hirth et al., 2000; EMA, 2011).

The comparison of expected utilities suggests that only for specific scenarios adaptive designs can be more efficient than fixed trial designs. Which design option is more attractive depends on the prevalence of the disease, the gains assigned to the possible outcomes of the trial and the prior distribution of the efficacy parameters in the different populations. Especially, only if there is a considerable uncertainty left regarding a homogeneity of the treatment effect across subpopulation the option to adapt the study population after an interim analysis can increase the efficiency of the trial.

## Supporting Information

As a service to our authors and readers, this journal provides supporting information supplied by the authors. Such materials are peer reviewed and may be re-organized for online delivery, but are not copy-edited or typeset. Technical support issues arising from supporting information (other than missing files) should be addressed to the authors.

## References

[b1] Alosh M, Huque M (2009). A flexible strategy for testing subgroups and overall populations. Statistics in Medicine.

[b2] Bauer P, Kieser M (1999). Combining different phases in the development of medical treatments within a single trial. Statistics in Medicine.

[b3] Bauer P, Koehne K (1994). Evaluations of experiments with adaptive interim analysis. Biometrics.

[b4] Bauer P, Koenig F (2014). The risks of methodology aversion in drug regulation. Nature Reviews Drug Discovery.

[b5] Beckman R, Clark J, Chen C (2011). Integrating predictive biomarkers and classifiers into oncology clinical development programmes. Nature Reviews Drug Discovery.

[b6] Boessen R, van der Baan F, Groenwold R, Egberts A, Klungel O, Grobbee D, Knol M, Roes K (2013). Optimizing trial design in pharmacogenetics research: comparing a fixed parallel group, group sequential, and adaptive selection design on sample size requirements. Parmaceutical Statistics.

[b7] Brannath W, Zuber E, Branson M, Bretz F, Gallo P, Posch M, Racine-Poon A (2009). Confirmatory adaptive designs with Bayesian decision tools for a targeted therapy in oncology. Statistics in Medicine.

[b8] Bretz F, Koenig F, Brannath W, Glimm E, Posch M (2009). Adaptive designs for confirmatory clinical trials. Statistics in Medicine.

[b9] Chen C, Beckman R (2009). Hypothesis testing in a confirmatory Phase III trial with a possible subset effect. Statistics in Biopharmaceutical Research.

[b10] Eichler H-G, Bloechl-Daum B, Brasseur D, Breckenridge A, Leufkens H, Raine J, Salmonson T, Schneider C, Rasi G (2013). The risks of risk aversion in drug regulation. Nature Reviews Drug Discovery.

[b11] EMA European Medicines Agency (2010). http://www.ema.europa.eu/docs/en_GB/document_library/Scientific_guideline/2010/05/WC500090116.pdf.

[b12] EMA European Medicines Agency (2011). http://www.ema.europa.eu/docs/en_GB/document_library/Report/2011/09/WC500112088.pdf.

[b13] Freidlin B, Korn E (2014). Biomarker enrichment strategies: matching trial design to biomarker credentials. Nature Reviews Clinical Oncology.

[b14] Freidlin B, McShane LM, Korn EL (2013). Randomized clinical trials with biomarkers: design issues. Journal of the National Cancer Institute.

[b15] Freidlin B, Simon R (2005). Adaptive signature design: an adaptive clinical trial design for generating and prospectively testing a gene expression signature for sensitive patients. Clinical Cancer Research.

[b16] Friede T, Parsons N, Stallard N (2012). A conditional error function approach for subgroup selection in adaptive clinical trials. Statistics in Medicine.

[b17] Hirth R, Chernew M, Miller E, Fendrick A, Weissert W (2000). Willingness to pay for a quality-adjusted life year in search of a standard. Medical Decision Making.

[b18] Hochberg Y (1988). A sharper Bonferroni procedure for multiple tests of significance. Biometrika.

[b19] Hommel G (2001). Adaptive modifications of hypotheses after and interim analysis. Biometrical Journal.

[b20] Jenkins M, Stone A, Jennison C (2011). An adaptive seamless phase II/III design for oncology trials with subpopulation selection using correlated survival endpoints. Pharmaceutical Statistics.

[b21] Jiang W, Freidlin B, Simon R (2007). Biomarker-adaptive threshold design: a procedure for evaluating treatment with possible biomarker-defined subset effect. Journal of the National Cancer Institute.

[b22] Kelly PJ, Stallard N, Todd S (2005). An adaptive group sequential design for phase II/III clinical trials that select a single treatment from several. Journal of Biopharmaceutical Statistics.

[b23] Lehmacher W, Wassmer G (1999). Adaptive sample size calculations in group sequential trials. Biometrics.

[b24] Magnusson BP, Turnbull BW (2013). Group Sequential enrichment design incorporating subgroup selection. Statistics in Medicine.

[b25] Maitournam A, Simon R (2005). On the efficiency of targeted clinical trials. Statistics in Medicine.

[b26] Mandrekar SJ, Sargent DJ (2009a). Clinical trial designs for predictive biomarker validation: one size does not fit all. Journal of biopharmaceutical statistics.

[b27] Mandrekar SJ, Sargent DJ (2009b). Clinical trial designs for predictive biomarker validation: theoretical considerations and practical challenges. Journal of Clinical Oncology.

[b28] Marcus R, Peritz E, Gabriel K (1976). On closed testing procedures with special reference to order analysis of variance. Statistics in Medicine.

[b29] Mehta C, Gao P, Bhatt DL, Harrington RA, Skerjanec S, Ware JH (2009). Optimizing trial design: sequential, adaptive, and enrichment strategies. Circulation.

[b30] Millen BA, Dmitrienko A, Ruberg S, Shen L (2012). A statistical framework for decision making in confirmatory multipopulation tailoring clinical trials. Drug Information Journal.

[b31] Mueller HH, Schaefer H (2001). Adaptive group sequential designs for clinical trials: combining the advantages of adaptive and of classical group sequential approaches. Biometrics.

[b32] Mueller HH, Schaefer H (2004). A general statistical principle for changing a design any time during the course of a trial. Statistics in Medicine.

[b33] Posch M, Bauer P (2013). Adaptive budgets in clinical trials. Statistics in Biopharmaceutical Research.

[b34] Sargent D, Mandrekar S (2013). Statistical issues in the validation of prognostic, predictive and surrogate biomarkers. Clinical Trials.

[b35] Simes RJ (1986). An improved Bonferroni procedure for multiple tests of significance. Biometrika.

[b36] Song Y, Chi GYH (2007). A method for testing a prespecified subgroup in clinical trials. Statistics in Medicine.

[b37] Stallard N, Hamborg T, Parsons N, Friede T (2014). Adaptive Designs for Confirmatory Clinical Trials with Subgroup Selection. Journal of Biopharmaceutical Statistics.

[b38] Stallard N, Posch M, Friede T, Koenig F, Brannath W (2009). Optimal choice of the number of treatments to be included in a clinical trial. Statistics in Medicine.

[b39] Wang SJ, O'Neill RT, Hung JHM (2007). Approaches to evaluation of treatment effect in randomized clinical trials with genomic subset. Pharmaceutical Statistics.

[b40] Wang SJ, Hung JHM, O'Neill RT (2009). Adaptive patient enrichment designs in therapeutic trials. Biometrical Journal.

[b41] Ziegler A, Koch A, Krockenberger K, Großhennig A (2012). Personalized medicine using DNA biomarkers: a review. Human Genetics.

